# JAK inhibitors in systemic juvenile idiopathic arthritis

**DOI:** 10.3389/fped.2023.1134312

**Published:** 2023-04-20

**Authors:** Tingyan He, Yu Xia, Ying Luo, Jun Yang

**Affiliations:** Department of Rheumatology and Immunology, Shenzhen Children’s Hospital, Shenzhen, China

**Keywords:** systemic juvenile idiopathic arthritis, JAK inhibitor, macrophage activation syndrome, tofacitinib, ruxolitinib

## Abstract

**Objective:**

Systemic juvenile idiopathic arthritis (SJIA) is characterized by excessive and inappropriate production of proinflammatory cytokines. Janus kinase inhibitors (JAKi) can block the downstream pathway of many cytokines. The use of JAKi in SJIA or macrophage activation syndrome (MAS) has only been described in a limited number of case reports. In this study, we aimed to assess the efficacy and potential adverse effects of JAKi in SJIA patients.

**Methods:**

Patients with SJIA who received JAKi and underwent at least one assessment of efficacy and safety after JAKi initiation were eligible for this study. Data were collected retrospectively from inpatient or outpatient medical records at JAKi initiation, at 1, 3, 6, 9, and 12 months, after disease flare, after JAKi discontinuation, or at the last follow-up.

**Results:**

Ten patients with SJIA were included in the study. At the start of JAKi treatment, all patients presented with active disease; five showed variable adverse effects secondary to glucocorticoids. Seven patients received tofacitinib (one later switched to ruxolitinib). Of these, only two patients showed a complete response of persistent arthritis associated with tocilizumab; tofacitinib was used without a biological DMARD only in two patients, together with MTX, showing a partial response; three patients were nonresponders. Four patients with SJIA-related MAS or persistent hyperferritinemia were treated with ruxolitinib. Ruxolitinib allowed a good response on MAS parameters in three of them. All these four patients required an adjunction or switch to canakinumab later. The median decrease in the daily glucocorticoid dose between JAKi initiation and the last follow-up was 90.6% in patients with complete remission and 77.4% in other patients. Three patients discontinued glucocorticoid treatment after the introduction of JAKi. Severe adverse events, notably serious infection or thrombosis, were not observed during JAKi treatment.

**Conclusion:**

JAKi may be an alternative or adjuvant agent for SJIA patients, especially in those with persistently active disease, glucocorticoid-related adverse reactions, or SJIA-MAS.

## Introduction

Systemic juvenile idiopathic arthritis (SJIA) is characterized by spiking fever, arthralgia or arthritis, myalgia, evanescent rashes, lymphadenopathy, hepatosplenomegaly, and serositis. SJIA-related macrophage activation syndrome (SJIA-MAS) is a life-threatening hyperinflammatory complication, exhibiting features such as fever, hyperferritinemia, thrombocytopenia, hypofibrinogenemia, hypertriglyceridemia, and elevated aspartate aminotransferase levels (1). Proinflammatory cytokines in SJIA-related MAS are excessively produced, including IFN-γ, TNF, IL-1, IL-2, IL-6, IL-10, IL-12, IL-18, and granulocyte macrophage-colony stimulating factor (GM-CSF). Notably, IL-18 is overproduced in an inflammasome-dependent manner by activated myeloid and epithelial cells. Excess-free IL-18 amplifies lymphocyte production of IFN-γ and has been proposed to contribute to MAS.

Janus kinase inhibitors (JAKi) interfere with the signal transduction pathways of various cytokines, such as IFN-γ, IL-2, IL-6, IL-10, IL-12, and GM-CSF (2). Tofacitinib, preferentially a JAK 1 and 3 inhibitor, can reduce the disease flare rate and is an effective treatment for patients with polyarticular course JIA (3). Ruxolitinib, as a JAK 1 and 2 inhibitor, has been tested in mouse models of hemophagocytic lymphohistiocytosis (HLH) and found to promote survival and reduce levels of proinflammatory cytokines IL-6 and TNF-α (4, 5). Ruxolitinib helped to control disease activity and reduce the cumulative dose of glucocorticoids in children with secondary hemophagocytic lymphohistiocytosis (6). Ruxolitinib combined with the doxorubicin–etoposide–methylprednisolone (Ru-DEP) regimen may be a safe and effective salvage therapy for refractory/relapsed HLH, especially MAS (7).

Although many patients respond to IL-1 or IL-6 inhibition, a subset of patients continue to have refractory SJIA or SJIA-MAS. Some patients develop persistent arthritis after resolving the systemic symptoms (8). A range of approaches for refractory SJIA-MAS is used, from a combination of steroids, IL-1 inhibitors, IL-6 inhibitors, and cyclosporine to etoposide with steroids to stem cell transplantation. Using JAKi in SJIA or SJIA-MAS has only been described in a limited number of case reports, showing a potential effect (9–11).

In this study, we described the largest cohort of SJIA patients receiving JAKi in the pediatric age to assess the efficacy and potential adverse effects of JAKi in patients with SJIA or SJIA-MAS.

## Methods

### Cohort of patients

Patients fulfilled the International League Against Rheumatism criteria for SJIA. SJIA patients who received JAKi and had at least one assessment of efficacy and safety after JAKi initiation were eligible for this study. Patients with the monogenic autoinflammatory syndrome were excluded by whole-exon sequencing. Concomitant treatment with glucocorticoids and/or other immunosuppressants was allowed.

### Data collection

Data were collected retrospectively from October 2018 to December 2022. Inpatient or outpatient medical records were reviewed at JAKi initiation, at 1, 3, 6, 9, and 12 months, after disease flare, after JAKi discontinuation, or the last follow-up. Data on clinical characteristics included systemic symptoms, skin rash, arthritis/arthralgia, hepatosplenomegaly, serositis, complications (MAS), and adverse effects (Tables 1, 2). Laboratory parameters included total leukocyte count, neutrophils, lymphocytes, hemoglobin, platelet count, erythrocyte sedimentation rate (ESR), C-reactive protein (CRP) level, serum aminotransferases, and ferritin and fibrinogen levels. Data on management [glucocorticoids, conventional synthetic or biological DMARDs (bDMARDs)] were collected.

**Table 1:** Clinical characteristics of SJIA patients at JAKi initiation.

Patients	Sex	Age at diagnosis (years)	Disease duration at initiation (years)	Age at initiation (years)	Main clinical manifestations at initiation	Laboratory characteristics at initiation	Treatments before JAKi initiation	Main reasons for JAKi initiation
P1	F	2	5	7	Arthritis	CRP↑	Steroids, MTX, tocilizumab	Active arthritis, growth retardation, hypertension, and cataract secondary to glucocorticoids
P2	F	6	7	13.7	Arthritis, frequent disease flare	WBC↑, CRP↑, ESR↑	Steroids, MTX, thalidomide, etanercept, cyclosporin, tocilizumab	Intolerance to tocilizumab, thoracic compression fracture, and growth retardation related to glucocorticoids
P3	M	7	0.05	7	MAS	ALT↑, AST↑, FER↑, FIB↓	Steroids, tocilizumab	No response to tocilizumab, severe intraocular hypertension secondary to glucocorticoids
P4	M	10.7	3	13	Fever, rash, arthritis	WBC↑, CRP↑, ESR↑, FER↑	Steroids, tocilizumab, MTX, anakinra, leflunomide, etanercept	Severe erythema at the anakinra injection site, insufficiency response to others
P5	M	14	0.5	14	Rash, arthritis	No	Steroids, tocilizumab, MTX	Active arthritis, hypertension secondary to glucocorticoids
P6	M	3.75	0.08	3.3	Fever, systemic edema, MAS	PLT↓, ALT↑, AST↑, FER↑, FIB↓	Steroids, tocilizumab	No response to tocilizumab, refractory MAS
P7	M	3	9	12.5	Fever, arthritis	WBC↑, FER↑, CRP↑	Steroids, tocilizumab, MTX, etanercept	Intolerance to tocilizumab, thoracic compression fracture and growth retardation related to glucocorticoids
P8	F	2.92	0.08	3	Fever, arthritis, MAS	WBC↑, FER↑, ALT↑, AST↑, FIB↓, ESR↑, CRP↑	Steroids	MAS
P9	F	7.66	8	16	Arthritis	ESR↑, CRP↑	Steroids, MTX, etanercept, tacrolimus, tocilizumab	Active arthritis
P10	M	11	2.5	13.4	Arthritis	WBC↑	Steroids, tocilizumab, MTX	Active arthritis

JAKi, Janus kinase inhibitors; WBC, white blood cells; FER, ferritin; MTX, methotrexate; CRP, C-reactive protein; ESR, erythrocyte sedimentation rate; ALT, alanine transaminase; AST, aspertate aminotranferase; FIB, fibrinogen; MAS, macrophage activation syndrome.

**Table 2:** Treatment of SJIA patients with JAK inhibitors.

Patients	Concomitant treatments at JAKi initiation	JAKi dosing	JAKi duration (months)	JAKi at the last follow-up	Response at JAKi duration	Treatments at the last follow-up	Remaining symptoms at last follow-up	Remaining abnormal laboratory findings at last follow-up	Infectious adverse effects at JAKi duration	Other adverse side effects at JAKi duration
P1	Prednisone (10 mg/day), MTX, tocilizumab	tofacitinib 2.5 mg bid → 3.75 mg bid	13	Discontinuation of JAKi for recurrent disease flare	No response, recurrent disease flare	Prednisone (2.5 mg/day), tocilizumab, adalimumab	Rash	CRP↑	Upper respiratory tract infection	No
P2	Prednisone (6.25 mg/day), MTX, NSAID	tofacitinib 2.5 mg bid → 10 mg bid	51	Ongoing	Partial response with mild disease flare	tofacitinib, MTX, NSAID	Occasional arthralgia	CRP↑	No	No
P3	Dexamethasone (15 mg/day)	ruxolitinib 2.5 mg bid → 5 mg bid	6	Ongoing	Partial response	Dexamethasone (0.375 mg/day), Canakinumab, ruxolitinib	No	No	No	No
P4	Methylprednisolone (8 mg/day), MTX	Tofacitinib 5 mg qd → ruxolitinib 7.5 mg bid	9	Ongoing	No response to the low dose of tofacitinib, partial response to ruxolitinib, disease flare	Dexamethasone (2.25 mg/day), canakinumab, ruxolitinib	Rash	FER↑	No	No
P5	Prednisone (15 mg/day), MTX, tocilizumab	tofacitinib 2.5 mg bid → 5 mg Bid	3	Discontinuation of JAKi for no response	No response, disease flare	Dexamethasone (0.75 mg/day), canakinumab	No	No	No	No
P6	Methylprednisolone pulse (25 mg/kg/day × 3 days) followed by Prednison (40 mg/day), etoposide	Ruxolitinib 2.5 mg bid → 5 mg bid	3	Discontinuation of JAKi for complete remission	Partial response, disease flare	Canakinumab, MTX	No	WBC↓	Upper respiratory tract infection	No
P7	Prednisone (12.5 mg/day), MTX	Tofacitinib 2.5 mg bid → 7.5 mg bid	27	Ongoing	Partial response, disease flare	Prednisone (7.5 mg/day), MTX, tofacitinib	Occasional fever and arthralgia	WBC↑, FER↑, CRP↑	No	No
P8	Methylprednisolone pulse (25 mg/kg/day × 3 days) followed by prednisone (25 mg/day), MTX, tocilizumab	Ruxolitinib 2.5 mg bid → 5 mg bid	4	Discontinuation of JAKi for complete remission	Complete response, rebound effect	Prednisone (1.25 mg/day), Canakinumab, MTX	No	No	Upper respiratory tract infection	No
P9	Prednisone (5 mg/day), MTX, tocilizumab	Tofacitinib 2.5 mg bid → 5 mg bid	24	Ongoing	Complete response with disease flare	MTX, tocilizumab, tofacitinib	No	No	No	No
P10	MTX, tocilizumab	Tofacitinib 5 mg qd → 5 mg bid	18	Ongoing	Complete response	Tocilizumab, tofacitinib	No	No	Skin abscess	No

JAKi, Janus kinase inhibitors; WBC, white blood cells; FER, ferritin; MTX, methotrexate; CRP, C-reactive protein; ESR, erythrocyte sedimentation rate; ALT, alanine transaminase; AST, aspertate aminotranferase; FIB, fibrinogen; MAS, macrophage activation syndrome.

### Definition of refractory SJIA and refractory SJIA-MAS

Patients with refractory SJIA are defined as patients with active disease who fail to respond to both IL-1 and IL-6 therapy and require maintenance therapy with glucocorticoids (12).

Refractory SJIA-MAS is defined as requiring long-term adjunctive therapy with GC or recurrent (≥2 episodes of) SJIA-related MAS.

### Response to JAKi treatment

Complete response was defined as the systemic Juvenile Arthritis Disease Activity Score (13) (sJADAS) <3 points and complete resolution of clinical SJIA-related symptoms and normalization of laboratory parameters, particularly inflammatory markers including total leukocyte count, ESR, CRP level, and ferritin level. Partial response was defined as decreased sJADAS but ≥3 points and with the persistence of some SJIA-related manifestations and/or elevation of inflammatory markers. No response was defined as the absence of clinically relevant improvement within 1–3 months of JAKi initiation. Disease flare was defined as the worsening of disease with elevated sJADAS, more SJIA-related symptoms, or abnormal laboratory values. The rebound effect was defined as a disease flare within 3 months after discontinuation of JAKi treatment.

### Study approval

The study was approved by the ethics committee of Shenzhen Children's Hospital. Written informed consent for the study and JAKi treatment was obtained from the legal guardians of all patients.

### Statistics

Data were expressed as median (minimal−maximal range). Analysis was performed with GraphPad Prism 8.0 statistical software (GraphPad Software Inc., La Jolla, CA, United States).

## Results

### Clinical characteristics of SJIA patients at disease onset and JAKi initiation

Ten SJIA patients who received JAKi treatment were identified at Shenzhen Children's Hospital; four were girls. The median age at diagnosis and JAKi initiation were 6.5 (2–11) years and 12.75 (3–16) years, respectively. At JAKi initiation, the median disease duration related to diagnosis was 2.75 (0.05–9) years (Table 1).

Before JAKi initiation, nine patients showed a failure of at least one bDMARD, and four had received more than one biological DMARDs. Tocilizumab was used in nine patients. Except for Patient 4 from Hong Kong, no other patients received IL-1 inhibitors since both anakinra and canakinumab were expensive and hard to get in the mainland of our country.

At the start of JAKi treatment, all patients presented with active disease [median sJADAS 14.1 (6, 28.2)]; five presented with various adverse effects secondary to glucocorticoids, including hypertension, thoracic compression fracture, cataract, severe intraocular hypertension, or growth retardation; and three new-onset patients (Patients 3, 6, and 8) had MAS; and nine had abnormal laboratory values related to disease activity (Table 1). At the start of JAKi initiation, nine patients were concomitantly treated with conventional synthetic and/or biological DMARDs, including five (Patients 1, 5, 8, 9, and 10) receiving tocilizumab; Patient 6 was concomitantly treated with etoposide.

### Efficacy of JAKi in SJIA

**Figure 1:**
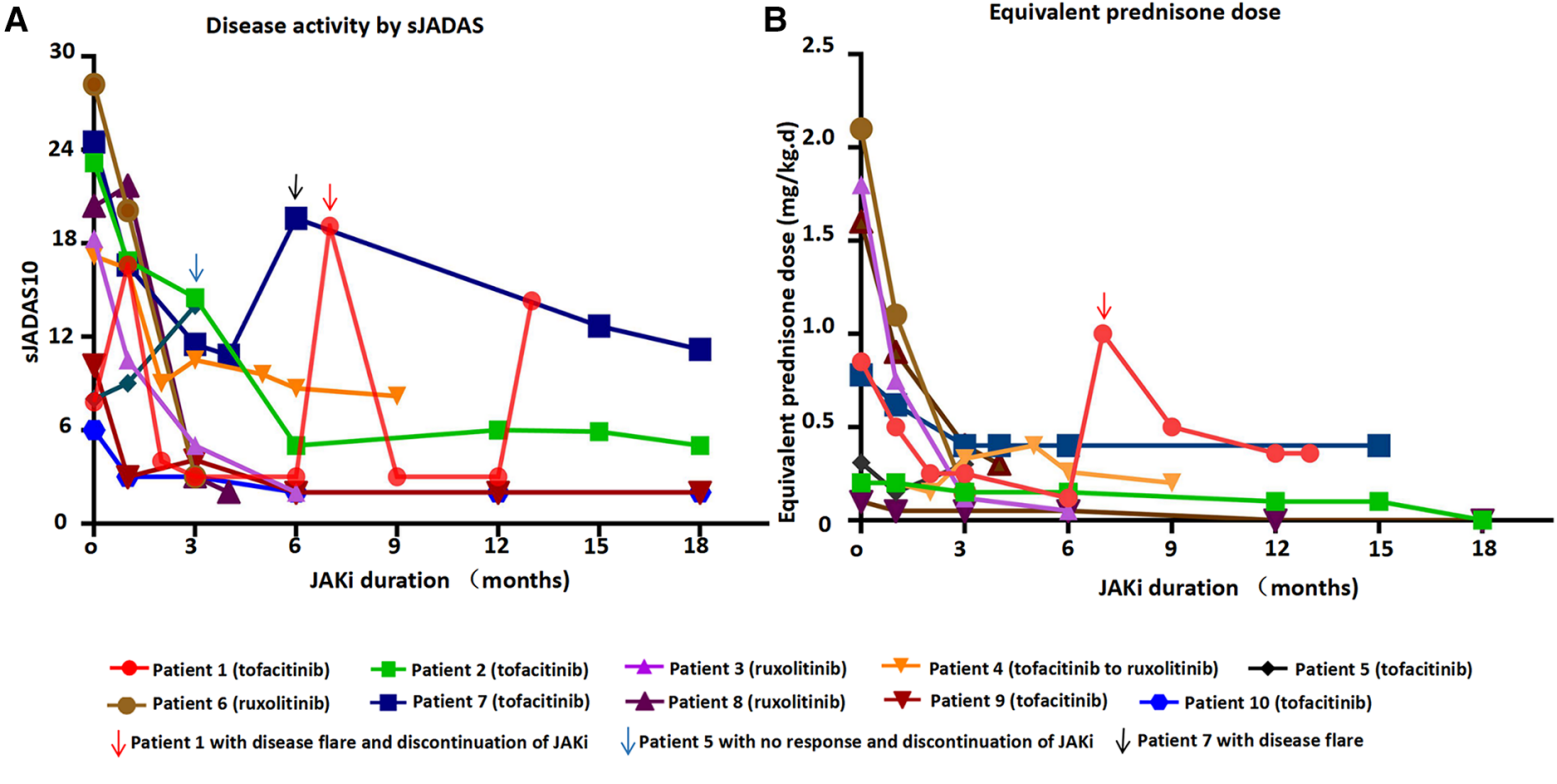
Disease activity by sJADAS and equivalent prednisone dose over time at JAKi duration.

Seven patients received tofacitinib after a median time of 5 years after the onset of the disease (Tables 1, 2 and Figure 1A). Tofacitinib was administered for a median time of 18 months (Table 2). Only Patients 9 and 10 showed a complete response of persistent arthritis in association with tocilizumab. Tofacitinib was used without a bDMARD in Patients 2 and 7 only, together with MTX, showing a partial response. Patient 2 showed great improvement in growth retardation with a height increasing from <5SD to <2SD. Three patients (Patients 1, 4, and 5) were nonresponders. Patient 1 discontinued JAKi treatment for recurrent disease flare and required a switch to anti-TNF treatment. Patient 4 switched to ruxolitinib and canakinumab for intermittent fever, persistent skin rash, and hyperferritinemia. Patient 5 showed no response to tofacitinib, presenting with persistent rash and carpal arthritis. When the dose of prednisone was reduced to 10 mg once a day, he presented with a disease flare characterized by fever, generalized rash, arthritis, and abnormal laboratory findings (leukocytosis, elevated ESR and CRP levels) (Figure 1B). After discontinuing tofacitinib treatment, he received canakinumab treatment and achieved a complete remission at the last follow-up.

Four patients with SJIA-MAS or persistent hyperferritinemia were treated with ruxolitinib (Tables 1, 2). Ruxolitinib was administered for a median time of 4 months. Patient 4 showed a partial response with residual manifestations of intermittent skin rash and hyperferritinemia. The other three patients (Patients 3, 6, and 8) showed a good response within 1 week after the introduction of ruxolitinib. Patient 3 presented with fever and other MAS-related manifestations after a slight reduction of glucocorticoids and showed no response to tocilizumab. Moreover, he suffered from severe intraocular hypertension 8 days after receiving high-dose glucocorticoids. He had a partial response to ruxolitinib, and the dose of glucocorticoids was gradually reduced to 42%. Patient 6 achieved partial remission for the first 11 days after the introduction of ruxolitinib, showing a complete resolution of fever and a reduction of serum ferritin level from 22,738 to 1,397 ng/ml. However, 1 week later, he presented with fever, systemic edema, and tachypnea. JAKi treatment was discontinued for 2 weeks to exclude any possible adverse effects and reintroduced considering it was the disease flare. Finally, as Patient 3, he achieved persistently complete remission after concomitant treatment of canakinumab. JAKi treatment was gradually discontinued after a total duration of 4 months. Patient 8 experienced MAS with a serum ferritin level as high as 34,471 ng/ml before ruxolitinib treatment. She achieved complete remission 1 month after concomitant treatment with glucocorticoids, ruxolitinib, MTX, and tocilizumab. After a complete resolution of 4 months, ruxolitinib was discontinued. Maintenance treatment included tocilizumab, MTX, and Prednisone 0.3 mg/(kg/day). However, she presented with a complete disease flare 2 months later after the discontinuation of ruxolitinib and was switched to canakinumab.

### Glucocorticoid-sparing effect

Nine patients received glucocorticoids at the start of JAKi treatment (Table 2 and Figure 1B). The median decrease in the daily glucocorticoid dose between JAKi initiation and the last follow-up was 90.6% in patients with complete remission and 77.4% in other patients. The glucocorticoid-sparing effect was not observed in Patient 5, who showed no response to JAKi. Three patients (Patients 2, 6, and 9) discontinued glucocorticoid treatment after introducing JAKi.

### Adverse effects of JAKi in SJIA patients

JAKi tolerance was good in all patients (Table 2). At JAKi duration, three had experienced upper respiratory tract infections and one suffered a skin abscess; one (Patient 6) showed persistent leukopenia even after discontinuation of JAKi treatment, more likely caused by myelosuppression secondary to etoposide. No patients stopped JAKi treatment for adverse effects. Severe adverse events, notably serious infection or thrombosis, were not observed during JAKi treatment.

## Discussion

The present cohort of 10 patients confirms the characteristic of SJIA, such as a high incidence of MAS complications, and severe adverse effects of glucocorticoids in a subset of patients, especially those with persistently active disease. In our series, side effects secondary to glucocorticoids included hypertension, thoracic compression fracture, cataract, severe intraocular hypertension, and growth retardation.

For patients receiving tofacitinib in the cohort, two (2/7, 28.6%) achieved complete remission of persistent arthritis in association with tocilizumab, two (2/7, 28.6%) showed partial remission, and three (2/7, 42.8%) showed no response. Huang et al. (9) described a patient complicated with osteoporosis and vertebral compression fracture, showing a steady improvement of both arthritis and systemic features after receiving tofacitinib therapy. Zhang et al. (14) reported a patient with SJIA treated with sequential tocilizumab and tofacitinib, showing complete remission of clinical symptoms. Therefore, tofacitinib may be an adjunct therapeutic agent for SJIA patients, especially those with persistent arthritis.

As the favorable response to ruxolitinib in HLH (6, 15), three patients with SJIA-MAS showed a good response within 1 week after introducing this agent. One patient showed a partial response with residual manifestations of intermittent skin rash and hyperferritinemia. However, all these patients required an adjunction or switch to canakinumab later. Therefore, ruxolitinib may be useful to treat MAS but not enough to control underlying SJIA disease.

This case series showed a significant reduction in median daily glucocorticoid use after JAKi initiation. The efficacy and glucocorticoid-sparing effect of JAKi treatment have been reported in another case series, including seven patients with adult-onset Still's disease and two with SJIA (10). These two SJIA patients had successful glucocorticoid tapering and achieved complete remission. Another patient presented with SJIA-associated severe early-onset lung disease, having a good response to ruxolitinib (16). In line with reported cases, our cohort has further confirmed the potential glucocorticoid-sparing effect of JAKi in SJIA patients, especially in those with glucocorticoid dependence or glucocorticoid-related adverse reactions.

Apart from the efficacy of JAKi, the optimal therapeutic dose remains to be defined. The final median doses of tofacitinib and ruxolitinib in our series are 5 (3.75–10) mg and 5 (5–7.5) mg twice a day, respectively. The dose of individual JAKi in various immune-mediated inflammatory diseases has been addressed in adults (2). The dose of tofacitinib for rheumatoid arthritis and ulcerative colitis is 5 and 10 mg twice a day, respectively. The recommended dosing for ruxolitinib varies from 5 to 25 mg twice daily. An open-label study has reported a much better improvement in children with Aicardi–Goutières syndrome in a higher-daily-dose category of baricitinib than those in a lower-daily-dose category (17). The median dose of ruxolitinib for STING-associated vasculopathy with onset in infancy is around 1 mg/kg/day. The therapeutic dose of JAKi in four SJIA patients with good response is tofacitinib 5 mg twice a day, ruxolitinib 1 mg/kg/day or 30 mg/day, and baricitinib 8 mg/day (9, 10, 16). As in interferonopathies, there might also be a dose-dependent efficacy in SJIA. A higher dose of JAKi may help quite a subset of patients in our cohort to achieve a much better clinical response, especially those with active systemic disease. Further pharmacokinetic studies will help to better determine the optimal dose of each JAK inhibitor in SJIA.

A long-term follow-up (≥13 months) of five patients under JAKi treatment confirms the good tolerance of this agent in SJIA. Serious infections, including opportunistic infections, were not observed in our series nor were other adverse events such as malignancy, embolism, anemia, or cytopenias. Therefore, risks of nonmelanoma skin cancer, venous thromboembolism, and cardiovascular events due to JAKi treatment might be much lower in SJIA patients. BK viremia was reported to be found in half of the interferonopathy patients treated with baricitinib (18). Although BK virus status was not evaluated in our patients, their renal function was always normal. The safety of JAKi in children might differ from that in adults, especially in those aged >60 years. Well-designed clinical trials will further clarify the safety of JAKi in SJIA patients.

The limitations of this study are the small sample size in a single center and that it is retrospective. Some clinical and laboratory information could not be collected. Serum cytokine levels were not regularly measured. SJADAS was not evaluated at all time points. We could not draw any conclusion about the association between the efficacy of JAKi treatment and cytokine profile. Because all patients concomitantly received conventional synthetic and/or biological DMARDs, the effect of JAKi alone could not be precisely analyzed. The efficacy of JAKi on glucocorticoid reduction may be biased by concomitant treatment with MTX or bDMARDs. The JAKi duration of three patients is less than 6 months. Given the current controversy about the safety of JAKi agents, a longer follow-up will help unravel the risk in children with SJIA.

## Conclusion

JAKi may be an alternative or adjuvant agent for SJIA patients, especially in those with persistently active disease, glucocorticoid-related adverse reactions, or SJIA-MAS. A higher dose of JAKi might help SJIA patients to achieve a much better clinical response, particularly those with active systemic disease. The overall tolerance of JAKi in children with SJIA may be good. Additional studies are required to better determine the optimal dose of each JAK inhibitor in SJIA.

## Data Availability

The original contributions presented in the study are included in the article/Supplementary Material, further inquiries can be directed to the corresponding authors.
